# Microbiomics Revealed the Disturbance of Intestinal Balance in Rabbits with Diarrhea Caused by Stopping the Use of an Antibiotic Diet

**DOI:** 10.3390/microorganisms10050841

**Published:** 2022-04-20

**Authors:** Jie Wang, Siqi Xia, Huimei Fan, Jiahao Shao, Tao Tang, Li Yang, Wenqiang Sun, Xianbo Jia, Shiyi Chen, Songjia Lai

**Affiliations:** 1 Farm Animal Genetic Resources Exploration and Innovation Key Laboratory of Sichuan Province, Sichuan Agricultural University, Chengdu 611130, China; wjie68@163.com (J.W.); wqsun2022@126.com (W.S.); jaxb369@sicau.edu.cn (X.J.); chensysau@163.com (S.C.); 2 College of Animal Science and Technology, Sichuan Agricultural University, Chengdu 611130, China; xiasiqi2020@163.com (S.X.); fanhuimei1998@163.com (H.F.); shaojh1997@163.com (J.S.); m18483220592@163.com (T.T.); ylyang1226@163.com (L.Y.)

**Keywords:** Hyplus rabbits, bacterial community, stop using antibiotics, diarrhea, 16 S rRNA

## Abstract

The harmful effects of diarrhea on the growth performance of rabbits have been well documented, but the details of the potential mechanism of intestinal diarrhea when antibiotics are stopped are still unclear. Here, PacBio sequencing technology was used to sequence the full length 16S rRNA gene of the microbiota of intestinal content samples, in order to characterize the bacterial communities in the small intestine (duodenum and jejunum) and large intestine (colon and cecum) in normal Hyplus rabbits and those with diarrhea. The histopathological examination showed that intestinal necrosis occurred in different degrees in the diarrhea group, and that the mucosal epithelium was shed and necrotic, forming erosion, and the clinical manifestation was necrosis. However, the intestinal tissue structure of the normal group was normal. The results revealed that there were significant differences in bacterial communities and structure between the diarrhea and normal groups of four intestinal segments (*p* < 0.05). In general, 16 bacterial phyla, 144 bacterial genera and 22 metabolic pathways were identified in the two groups. Tax4Fun functional prediction analysis showed that KEGG related to amino acid metabolism and energy metabolism was enriched in the large intestines of rabbits with diarrhea, whereas lipid metabolism was more abundant in the small intestine of rabbits with diarrhea. In conclusion, the change in the relative abundance of the identified dominant microbiota, which could deplete key anti-inflammatory metabolites and lead to bacterial imbalance and diarrhea, resulted in diarrhea in Hyplus rabbits that stopped using antibiotics.

## 1. Introduction

The mammalian gut hosts complex microbial communities that maintain a symbiotic relationship with the host, which is not only necessary for immune homeostasis, metabolism, nutrition, and physiology, but also affects the host’s susceptibility to many immune mediated diseases and disorders [1]. In addition, the production traits of economic animals, such as rabbits, are also affected by intestinal microbiota [2]. A recent study has shown that the intestinal microbiome of rabbits plays an important role in regulating body weight [3]. As the accuracy of high throughput sequencing technologies continues to improve, so does the understanding of how microbial communities interact with their hosts [4]. With the advent of sequencing technology using PacBio, the sequencing of the full length of the 16S rRNA gene of microbiota has gradually emerged [5]. The identification of bacteria by 16S rRNA not only provides a comprehensive characterization of the microbe in a culture independent manner, but also provides additional relevant information about different taxa in the gut. Many countries have enacted laws prohibiting the use of antibiotic added diets, however, under such feeding conditions, animal mortality increases, breeding costs also increase, and the quality of animal products decreases [6,7]. The structural integrity of the gut mucosa is critical for the gut to function, leading to diarrheal diseases in livestock, such as epidemic necrotizing enteritis, following the ban on antibiotics in the European Union [8]. However, studies using PacBio sequencing of full length 16S rRNA on the gut microbiome of antibiotic free rabbits are very limited.

Diarrhea is a common disease in rabbits, especially young weaned rabbits, and its incidence is quite high [9]. When rabbits are suddenly forced to adapt to a series of factors such as nutrition and environmental interference, the intestinal microbial system will produce disorders, resulting in loss of appetite, growth retardation and diarrhea, and even death, so the rabbit breeding industry has experienced a lot of losses [10,11]. In other species, such as pigs [12], chickens [13], mice [14], calves [15], and others, previous studies have described differences in gut microbiota after diarrhea versus nondiarrhea groups. Generally speaking, it may be due to the increase in the abundance of harmful bacteria (*Escherichia coli*, *Clostridium* and *Bacteroides*) and the decrease in the abundance of beneficial bacteria (*Lactobacillus casei*, *Bifidobacterium* and *Lactobacillus*), so as to promote the release of proinflammatory signal factors (proinflammatory release cytokines, IL-6 and TNF-α, as well as the secretion of immunoglobulin A (IgA)) and reduce the level of short chain fatty acids (SCFAs), thereby inhibiting intestinal ion and water absorption, destroying intestinal permeability, producing mucosal inflammation. Finally, homeostasis is destroyed and diarrhea occurs [13,14,15,16]. The use of antibiotics in the animal feeds industry had been used for many years, not only to control disease, but also to boost feed efficiency and improve growth performance [17]. However, the intake of antibiotics by edible animals and the resulted residue of antibiotics in food have been considered to be an important reason for the rapid spread of antibiotic resistance (ABR) in the human population, as well as serious health problems worldwide [18]. Therefore, the reduction in antibiotic intake and residues in edible animals has played a central role in food safety and global public health threats. The prohibition of the use of growth-promoting drug additives in feeds was implemented in China in 2020. Previous studies in mice have shown that gut microbes do not immediately return to normal when antibiotics are stopped, and were limited to the large intestine or small intestine [19]. The content of amino acids (Anthranilic acid, L-Tryptophan, and L-Phenylalanine) in a diarrhea group of rabbits fed without antibiotics was higher than that in a normal group [20]. Previous studies have shown that the use of antibiotics changes the link between intestinal microbes and amino acid metabolism [21]. After the relative abundance of *Proteobacteria* changes, it will cause a decrease in alanine and branched chain amino acids [22]. In addition, the expression profile of the host transcriptome of rabbits fed without antibiotics changed, and the differentially expressed genes may be related to model inflammation, adaptive immune response, etc. [23]. The intestinal flora of animals is necessary for the conversion of bile acids: a previous study found that the level of lithocholic acid produced by bile acid metabolism was significantly reduced in a group with diarrhea; lithocholic acid has a protective effect on the intestinal epithelial barrier and may be related to the pathogenesis of inflammatory bowel disease, meaning that metabolites and gut microbes interact [24]. Antibiotic free rabbits developed intestinal dysbiosis, the relative abundance of reported protective species was reduced, and the richness, uniformity, and diversity of gut bacterial communities were significantly reduced, creating a distinct taxonomy and inferred dysfunction [25]. However, the mechanism by which intestinal microorganisms produce diarrhea in the large and small intestine after discontinuation of antibiotics in rabbits is unclear.

Here, we characterized the changes in intestinal microbiome in normal rabbits and those with diarrhea after stopping using antibiotic by 16S rRNA amplification sequencing. Notably, we studied the lumen contents of four intestinal segments simultaneously, rather than a single segment. These results will be helpful for the pathogenesis and treatment of diarrhea in rabbits, so as to improve the production performance of rabbits.

## 2. Materials and Methods

### 2.1. Ethics Statement

This study was approved by and conducted in strict accordance with the ethical standards of the Institutional Animal Care and Use Committee of the College of Animal Science and Technology, Sichuan Agricultural University, Ya’an, Sichuan, China (DKY-B20090908).

### 2.2. Histological Observation

To observe histological changes in the four intestinal segments (FIS) (including cecum, colon, duodenum, and jejunum tissue), Hyplus rabbits were chosen to be humanely slaughtered and stained with hematoxylin-eosin. Briefly, intestinal tissue samples were fixed in 10% neutral formaldehyde for 24 h and then washed with water. Then, FIS tissue samples were dehydrated, paraffin embedded, and stained with hematoxylin-eosin. FIS tissue sections (5 μm) were collected using microtome (Leica RM2235, Wetzlar, Germany). After that, pictures were taken at 100× using a light microscope (OLMPUS CX22, Tokyo, Japan) with an imaging system (Leica DM1000, Wetzlar, Germany). 

### 2.3. Experimental Design and Samples Collection

**Figure 1:**
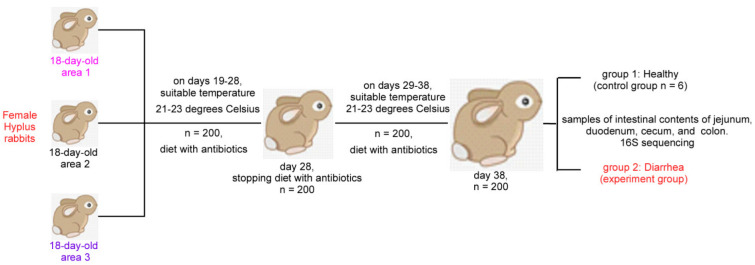
Overview of workflow for comprehensive analysis of intestinal microbiota of Hyplus rabbits of the same age group.

In this study, two hundred 38-day-old female Hyplus rabbits from three areas of Zhongtian rabbit farm were selected. The whole experimental workflow is shown in Figure 1. These rabbits were fed a diet containing antibiotics for 10 days before weaning and had stopped using the diet containing antibiotics for 10 days after weaning. All rabbits were raised under standard farm management conditions and were routinely vaccinated. At the end of the experiment, 6 rabbits from the normal condition were selected as the control group (Con, *n* = 6) and 6 rabbits from the disease condition (diarrhea) were selected as the experiment (diarrhea) group (Dia, *n* = 6) according to their weight, physiological condition and health status [26]. Each rabbit was fed 150–200 g diet per day and lived separately in a clean cage (600 × 600 × 500 mm, 21–23 °C, 60–75 % humidity, 14 h light (60 lx)) [20]. These animals had free access to fresh water. After 24 h without food, 12 rabbits were euthanized under anesthesia. Samples of the contents of the FIS (including cecum, colon, duodenum, and jejunum contents) were immediately collected in 2 mL freezing tubes, which were frozen in liquid nitrogen and stored at −80 °C for subsequent tests. 

### 2.4. DNA Extraction and 16S rRNA Gene Sequencing

Microbiome DNA from FIS contents were extracted using the Cetyl trimethyl ammonium bromide (CTAB)/SDS method according to a previously described protocol [27], and the purity and concentration of DNA were detected by agarose gel electrophoresis. Using diluted genomic DNA (1 ng/μL) as template, specific primers with the barcode (Forward (5′-3′): AGAGTTTGATCCTGGCTCAG, Rreverse (3′-5′): GNTACCTTGTTACGACTT) were amplified from full-length 16S rRNA genes. TransStart^®^ FastPfu DNA Polymerase (TransGen Biotech Co., LTD, Bejing, China) was used in all PCR reactions in our study. The PCR products were detected by electrophoresis using 2% agarose gel, and then the products were purified with QIAquick Gel Extraction Kit (Qiagen, Hilden, Germany). According to the manufacturer’s instructions, the DNA library was prepared using SMRTbellTM Template Prep Kit (Pacific BioSciences, Inc., Menlo Park, CA, USA) and then sequenced on the PacBio platform (Pacific BioSciences, Inc., Menlo Park, CA, USA).

### 2.5. Sequencing Data Analysis

In order to obtain effective data, CCS (SMRT Link V7.0) was used for sequence correction of original data to remove unqualified sequences (min length: 1340 bp, max length: 1640 bp), followed by SSR filtration and primers removal [28]. Before subsequent analysis, Uparse (V.7.0.1001, http://drive5.com/uparse/, accessed on 8 December 2020) [29] was used to cluster the sequences with 97% identity to become OTUs (operational taxonomic units). Species annotation analysis was performed on the representative sequences of OTUs based on the SSUrRNA database of the Silva database (http://www.arb-silva.de/, accessed on 8 December 2020) (threshold: 0.8–1) [30] with the Mothur (V.1.41.1) algorithm, and the taxonomic information of each sample, such as phylum, genus and species, was obtained. Before diversity analysis, the OTUs’ abundance information was normalized with the sequence standard corresponding to the sample with the least sequence divergence. QIIME (V.1.9.1) was used to calculate alpha (Observed_species, Shannon index, Chao1 index, and PD_whole_tree) and beta diversity [3], and rarefaction curves, rank abundance were displayed with R (V.2.15.3). The species complexity differences of the samples were evaluated by beta diversity analysis, and then the beta diversity on the weighted UniFrac metrics was calculated by QIIME (V.1.9.1). Principal component analysis (PCA) was performed first, and then the original variables were dimensionally reduced using the FactoMine and ggplot2 package of R software (Version 2.15.3). The visualization results of PCoA analysis were displayed using WGCNA, stat and ggplot2 packages of R software (Version 2.15.3). Linear discrimination analysis (LDA) effect size (LEfSe) analysis used LEfSe software (LDA Score ≥ 4). Analysis of molecular variance (AMOVA) was analyzed using the Mothur software (V.1.41.1) [31]. The relative column accumulation diagram was produced using the bacterial community annotation results to select the highest abundance (top 10, 20, 30, respectively) at each taxonomic level (phylum, genus, species) for each sample or group, in order to show the relative abundance ratio. 

### 2.6. Tax4Fun Prediction of the Microbes in FIS’s Contents

The functional characteristics of gut microbial community function were predicted from 16S rRNA sequence data using the Tax4Fun software package [32]. In the present study, functional prediction was based on OTU richness, as previously described. Then, these predicted functional profiles were aggregated to Kyoto Encyclopaedia of Genes and Genomes (KEGG) from the first, second, and third levels, respectively. Finally, we selected the functional spectrum of the third level for z-score normalization to generate a cluster heatmap. 

### 2.7. Statistical Analysis

In this study, Student’s test, LEfSe test, AMOVA, and Kruskal–Wallis test were used to compare different FIS or groups. Here, *p* < 0.05 was considered statistically significant. Group means were expressed as the mean ± standard error (mean ± SE).

## 3. Results

### 3.1. Histological Observation of FIS

**Figure 2:**
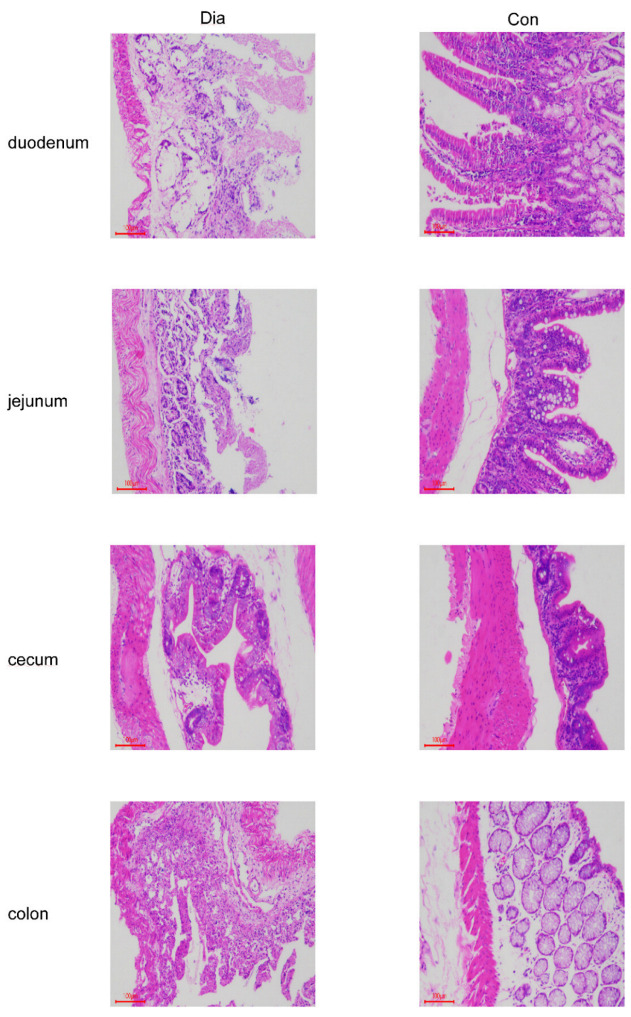
Hematoxylin and eosin stained duodenal, jejunum, cecum, and colon tissue samples from Dia (*n* = 6) and Con (*n* = 6) Hyplus rabbits caused by stopping the use of antibiotic diet. Con, control group; Dia, diarrhea group.

The histopathological examination of the FIS showed different grades of necrosis in the intestinal tissue in Dia (Figure 2). In addition, the intestinal mucosa of the Dia also had necrosis and shedding of the intestinal mucosa, resulting in erosion, and, finally, diarrhea. However, in the Con, the intestinal tissue structure was normal, and no obvious histopathological damage was found. Our study showed that stopping antibiotics had a certain effect on the intestinal morphology of rabbits.

### 3.2. Overview of Sequence Data Analysis

After filtering out sequences that were too long or too short (ranged: 1426–1471) and removing primers and simple sequence repeats (SSR), a total of 439,610 clean reads were obtained from all samples, and the average clean reads per sample was 9159 ± 962 (mean ± SE) (Table S1). The average sequence length of all the samples was 1451 ± 1.6 (mean ± SE), and the sequence length of effective rate was 86.03 ± 0.48 (%) (Table S1). We used 97 percent of the identity and OTUs in the range of 37–1629 were identified in all samples. The rarefaction curves indicate that the curve tends to be horizontal, in general (Figure S1A), indicating that the current sequencing volume saturation is sufficient. In addition, we obtained the species richness curve, which can directly reflect the richness and evenness of bacterial groups in the samples (Figure S1B). 

### 3.3. Analysis of Alpha Diversity of Bacteria in FIS

**Figure 3:**
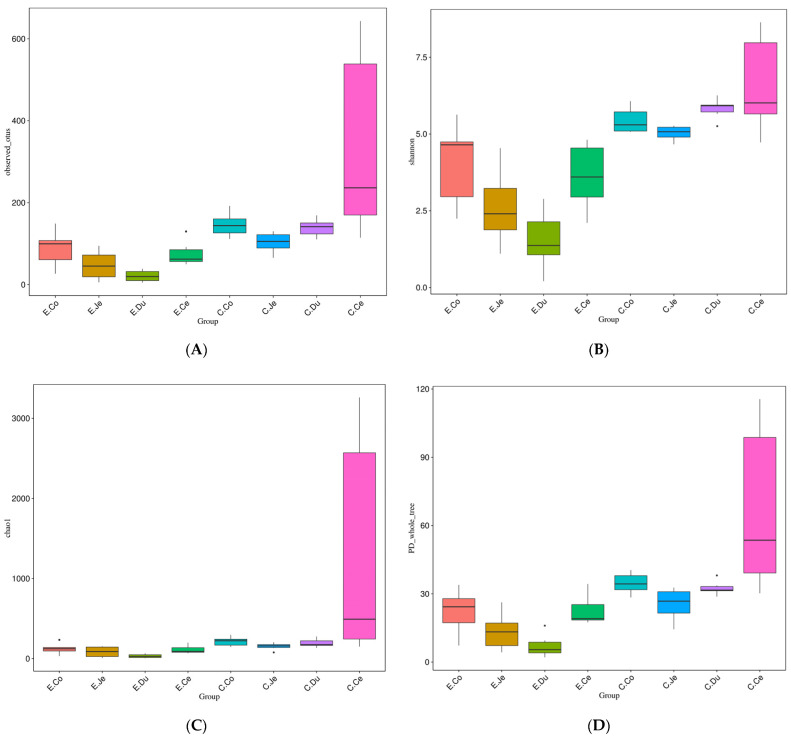
Analysis of alpha diversity index of bacteria of four intestinal segments (FIS) in Dia (*n* = 6) and Con (*n* = 6) Hyplus rabbits caused by stopping the use of antibiotic diet. (**A**) Observed_OTUs (species) in the four intestinal regions. (**B**) Shannon index of the four intestinal regions. (**C**) Chao1 index of the four intestinal regions. (**D**) PD_whole_tree of the four intestinal regions. C, control group (Con, *n* = 6); E, diarrhea group (Dia, *n* = 6), Co, colon; Ce, cecum; Du, duodenum; Je, jejunum.

The bacterial full length 16S rRNAs were sequenced to analyze the microbial community in the intestinal contents of FIS in Con and Dia. The biodiversity index of the bacterial community were analyzed, respectively. The results showed that the distribution of the biodiversity index in the different intestinal segments of the two groups was diverse. For instance, the four diversity indexes (Observed_species (Observed_OTUs), Shannon index, Chao1 index, and PD_whole_tree) were all significantly dissimilar in the FIS of each group (*p* < 0.05) (Table 1). In Dia, a higher diversity index was observed in the colon and a lower diversity index was observed in the duodenum. There were significant differences in the four diversity indexes between the large intestine and the duodenum (*p* < 0.05). However, there were no significant differences in the four diversity indexes between the cecum and colon, between the cecum and jejunum, nor between the duodenum and jejunum (*p* > 0.05). In the Con, a higher diversity index was observed in the cecum and a lower diversity index was observed in the jejunum. There were significant differences in Observed_species, Shannon index, and PD_whole_tree between the cecum and jejunum (*p* < 0.05). However, there were no significant differences in Observed_species, Chao1 index, and PD_whole-tree between the colon and duodenum (*p* > 0.05). Additionally, the PD_whole-tree between the duodenum and jejunum was significantly different (*p* > 0.05). The alpha diversity of the FIS in the Con and Dia was compared, and the observed values of the FIS in Con were all more abundant than those in Dia (Figure 3). As reflected by the four indexes, we observed that diarrhea reduces the alpha diversity of the gut microbial community. 

### 3.4. Analysis of Beta Diversity of Bacteria in FIS

**Figure 4:**
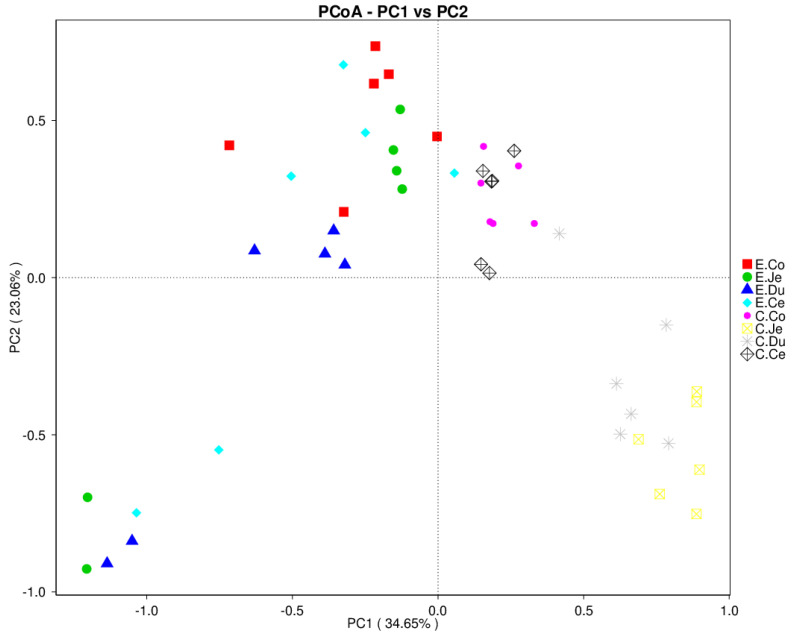
Analysis of beta diversity index of bacteria in four intestinal segments (FIS). Principle coordinates analysis (PCoA) of bacterial communities in FIS of Dia (*n* = 6) and Con (*n* = 6). The PCoA of bacterial communities in all samples based on weighted Unifrac. C, control group (Con, *n* = 6); E, diarrhea group (Dia, *n* = 6), Co, colon; Ce, cecum; Du, duodenum; Je, jejunum.

Principal coordinates analysis (PCoA) based on weighted Unifrac was used to assess the structure of bacterial communities in the FIS of the Con and Dia, and the results showed that the structures in the small and large intestine were different, and the two groups were also distinct (Figure 4). AMOVA was used to examine the significance of differences in bacterial communities between Con and Dia. A weighted UniFrac distance matrix was also used to test the significance of differences between the two groups. The results indicated no significant differences between the cecum and colon (*p* = 0.43) in Con, and between the duodenum and jejunum (*p* = 0.152), cecum and jejunum (*p* = 0.398), and cecum and colon (*p* = 0.086) in Dia (Table S2). However, the difference between the other segments in the two groups was significant (*p* < 0.05). Furthermore, we also observed that, based on weighted UniFrac distance, there was an obvious discrepancy in the bacterial structure of the FIS between Con and Dia (*p* < 0.05) (Table S3).

### 3.5. Distribution of Main Bacteria in the Intestinal Tract of Hyplus Rabbits

**Figure 5:**
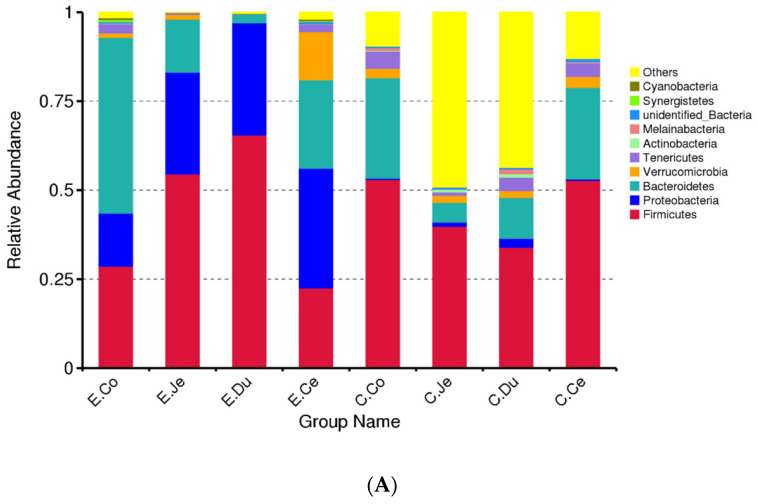

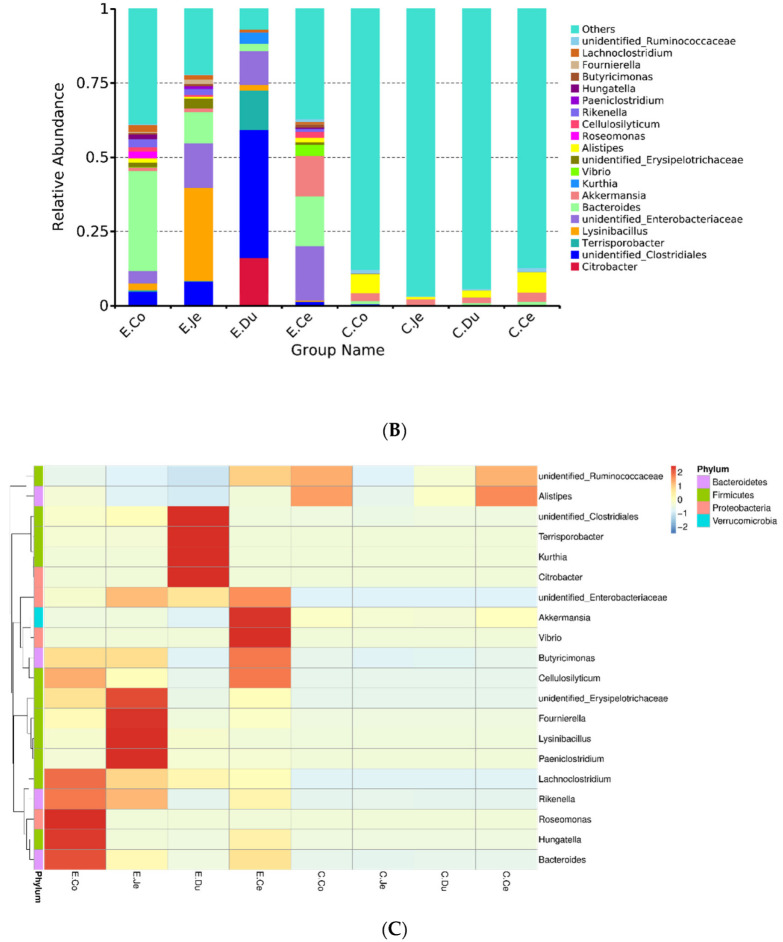

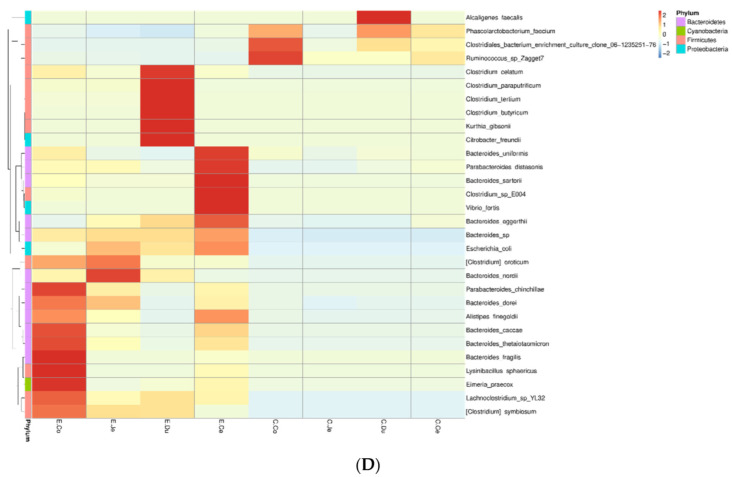
The relative abundances of dominant bacteria in the FIS of the Con (*n* = 6) and Dia (*n* = 6). (**A**) The top ten most abundant species at the phylum taxonomic level of each sample generated as a columnar cumulative plot of relative abundance of species. (**B**) The top 20 species with the highest abundance at the genus taxonomic level in each group generated as a columnar cumulative plot of relative abundance. (**C**) Heat maps of relative abundances of the top 20 dominant bacteria genera with the highest intestinal richness in Hyplus rabbit. (**D**) Heat maps of relative abundances of the top 30 dominant bacteria species with the highest intestinal richness in Hyplus rabbit. C, control group (Con, *n* = 6); E, diarrhea group (Dia, *n* = 6); Co, colon; Ce, cecum; Du, duodenum; Je, jejunum.

At the phylum level, the bacterial communities in the FIS were mainly *Bacteroidetes* (23.62%), *Firmicutes* (42.38%), and *Proteobacteria* (24.78%) (Figure S2). The top ten most abundant phyla in each group were selected to generate a column accumulation chart of relative abundance (Figure 5A), including *Firmicutes*, *Proteobacteria*, *Bacteroidetes*, *Verrucomicrobia*, *Tenericutes*, *Actinobacteria*, *Melainabacteria*, *unidentified_Bacteria*, *Synergistetes*, and *Cyanobacteria*. However, the relative abundance of these phyla was dissimilar in the FIS (Table S4). For instance, *Firmicutes*, *Proteobacteria* and *Bacteroidetes* were the dominant in the FIS, and these dominant bacteria were different between the FIS and the two groups. Additionally, compared with the Con, the average relative abundance of *Firmicutes* decreased in large intestine, whereas the other two dominant bacteria (*Proteobacteria* and *Bacteroidetes*) increased in the FIS, and the relative abundance of *Firmicutes* is lower in the large intestine in Dia (Table S4). The relative abundances of *Tenericutes* and *Melainabacteria* were also lower in the Dia than in Con. *Melainabacteria* was not detected in the jejunum of Dia, while it was detected in the Con (Table S4). We also observed that the abundance of *Bacteroidetes* in the duodenum was the lowest in the Dia (Table S4). Finally, we also found that *Synergistetes* were detected only in the large intestine of the Dia (Table S4). According to the significant differences between the FIS of the two groups, two phyla were selected in the Dia, including *Firmicutes* and *Bacteroidetes* (*p* < 0.05), and three phyla were selected in the Con, including *Firmicutes*, *Bacteroidetes* and *Tenericutes* (*p* < 0.05) (Table S4). *Firmicutes* and *Bacteroidetes* showed significant differences among the FIS (*p* < 0.05), while the other eight phyla were not significant (*p* > 0.05) (Table S4). 

At the genus level, a total of 144 genera were identified from the FIS in both groups (Table S5). The top 20 most abundant bacteria genera in the two groups were selected to generate a column accumulation chart of relative abundance (Figure 5B). In the FIS, the relative abundance of the dominant genera (only genera with a relative abundance ≥5% in at least one of the four segments were shown) was ten in Dia and five in Con (Table S6). The dominant genera in Dia included *Citrobacter*, *Unidentified_clostridiales*, *Terrisporobacter*, *Lysinibacillus*, *unidentified_Enterobacteriaceae*, *Bacteroides*, *Akkermansia*, *Kurthia*, *Vibrio* and *unidentified_Erysipelotrichaceae* (Table S6). In addition, the dominant genera in the Con included *unidentified_Clostridiales*, *unidentified_Enterobacteriaceae*, *Bacteroides*, *Akkermansia*, and Vibrio (Table S6). However, the relative abundances of these genera were dissimilar in the FIS (Table S6). The top 20 genera in abundance were selected to cluster at both the genus and sample levels, and the heat map was drawn (Figure 5C). Results showed that the bacterial communities in the intestinal regions of the Con and Dia were distinct from each other. 

In addition, we selected bacterial species with relative abundances of ≥5% in each of the FIS, and found the dominant bacteria in Dia to include *Clostridium_butyricum,* and *Escherichia_coli*, and the relative abundance in Dia was higher than that in Con. The top 30 species in abundance were selected to cluster at both the species and sample levels, and the heat map was also drawn (Figure 5D). Results showed that the bacterial communities in the intestinal of the Con and Dia were distinct from each other.

### 3.6. Changes in Intestinal Bacteria in FIS

**Figure 6:**
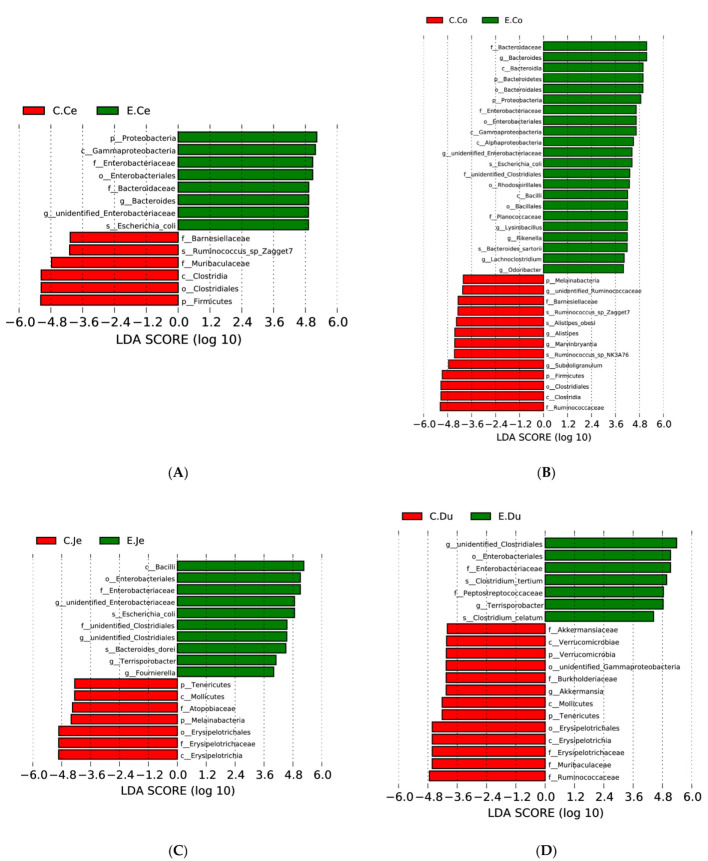
Linear discriminant analysis (LDA) effect size analysis of FIS in Hyplus rabbits (Con, *n* = 6; Dia, *n* = 6). LDA score ≥ 4 demonstates a biomarker with statistical differences between Con and Dia groups. (**A**) shows the differentially expressed flora in the cecum of the Dia group and the cecum of the Con group; (**B**) shows the differentially expressed flora in the colon of the Dia group and the colon of the Con group; (**C**) shows the differentially expressed flora in the jejunum of the Dia group and the jejunum of the Con group; (**D**) shows the differentially expressed flora in the duodenum of the Dia group and the duodenum of the Con group; p: phylum, c: class, o:order, f:family; g: genus; s:species. C, control group (Con, *n* = 6); E, diarrhea group (Dia, *n* = 6); Co, colon; Ce, cecum; Du, duodenum; Je, jejunum.

Next, we performed LEfSe to identify discriminatory biomarkers (LDA scores ≥ 4) in the FIS between Con and Dia (Figure 6A–D). At the same time, the diversity analysis results were also used to screen the discriminatory biomarkers. The discriminant biomarkers enriched in Con included members from the phylum *Firmicutes*, while in Dia they included members from the phylum *Proteobacteria* and *two* members from the genus *unidentified_Enterobacteriaceae* and *Bacteroides*, and the specie *Escherichia_coli* of the cecum. Moreover, the discriminant biomarkers enriched in Con included two members from the phylum *Firmicutes* and *Melainabacteria*, while in Dia they included members from the phylum *Proteobacteria* and three members from the genus *unidentified_Enterobacteriaceae*, *Bacteroides* and *Lysinibacillus* of the colon. In addition, the discriminant biomarkers enriched in Con included two members from the phylum *Tenericutes* and the genus *Akkermansia*, while in Dia they included members from genus *Unidentified_clostridiales, Terrisporobacter* of the duodenum. Furthermore, the discriminant biomarkers enriched in Con included a member from the phylum *Melainabacteria*, while in Dia they included members from three genus, *Unidentified_clostridiales*, *Terrisporobacter* and *unidentified_Enterobacteriaceae*, of the jejunum. 

### 3.7. Predictive Functional Pathway Analysis of the FIS

At the first level, functional prediction in the FIS contained cellular processes, environmental information processing, genetic information processing, human diseases, metabolism, and organismal systems (Table S7). Cellular processes and metabolism had significant differences in the FIS of the Dia group (*p* < 0.05) and no significant differences in the Con group (*p* > 0.05), and other functions had no significant differences in the FIS of Con and Dia (*p* > 0.05) (Table S7).

At the second level, this study enriched 15 functional pathways in the FIS (the relative abundances of these pathways were greater than 5% in at least one segment in both groups) [33]. Using the Kruskal–Wallis test, we found that the relative abundance (transport and catabolism, folding, sorting and degradation, metabolism of cofactors and vitamins, lipid metabolism, glycan biosynthesis and metabolism, energy metabolism, and amino acid metabolism) were higher in the large intestine than in the duodenum of Dia, and the differences between these intestinal segments were significant (*p* < 0.05). In addition, the relative abundance of the metabolism of cofactors and vitamins was higher in the jejunum than in the duodenum, and the difference between them was significant (*p* < 0.05). Moreover, compared with the cecum, the relative abundance of lipid metabolism was more abundant in the colon, and the difference between them was also significant (*p* < 0.05). Furthermore, the relative abundance of amino acid metabolism was low in the jejunum; nevertheless, it was abundant in the the cecum and colon, and there was a significant difference between the cecum, colon and jejunum (*p* < 0.05). However, these findings were only concentrated in the Dia, and no similar results were found in the Con. However, only the three metabolic pathways, including amino acid metabolism, energy metabolism and lipid metabolism, showed significant differences between Dia and Con in the large or small intestines (*p* < 0.05)

**Figure 7:**
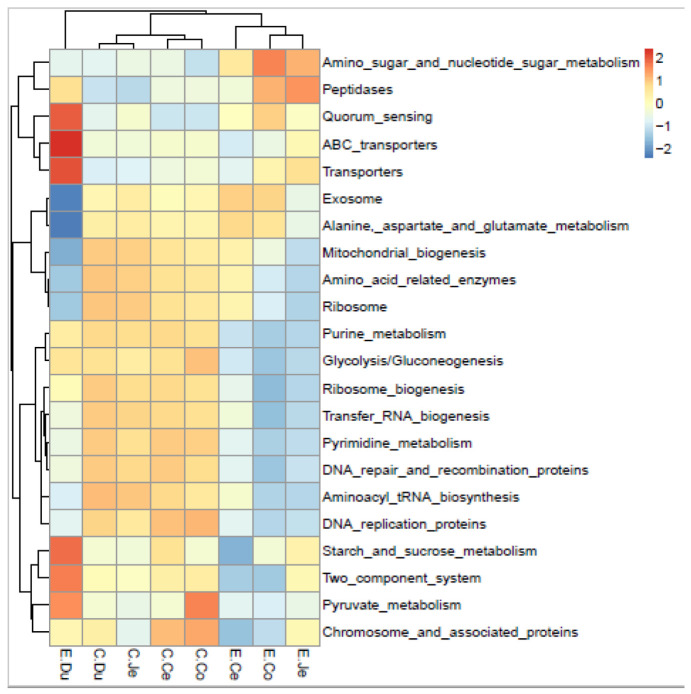
Heatmap of relative abundance of the dominant KEGG orthologs (KOs) pathway at the third level (the pathway with average relative abundance ≥1% in at least one region in FIS of Hyplus rabbits). C, control group (Con, *n* = 6); E, diarrhea group (Dia, *n* = 6); Co, colon; Ce, cecum; Du, duodenum; Je, jejunum.

At the third level, 22 KEGG orthologs (KOs) pathways were selected (mean relative abundance ≥1% in at least one intestinal segment from the two groups) from the total pathways (Figure 7). The results of the Spearman distance matrix evaluation of the relative abundance and the heat map of these 22 pathways showed that the intestinal segments in the Con and Dia were distinct, and the large and small intestine in the Con were distinct, while the colon and jejunum were clustered in the Dia. In addition, the duodenum and the other three segments of the intestine were not clustered together. 

## 4. Discussion

In our study, we identified bacterial communities in the FIS of Con and Dia, and sequenced the full length of the 16S rRNA gene. The aim of this study was to understand the distribution and underlying functions of the microbiota associated with FIS’s contents in the Dia and Con rabbits, mainly focusing on the microbiota differences between the large and small intestines. The rarefaction curves and species richness curve analysis results showed that the current sampling and sequencing results can be used to analyze the bacterial community. 

The diversity of bacteria in the large intestine was higher than those in the small intestine; the diversity index of the colon was the highest and that of the duodenum was the lowest in the Dia, while the diversity index of the cecum was the highest and that of the jejunum was the lowest in the Con. The results are consistent with a previous study that reported up to 1000 species of bacteria in the human colon, while the species richness and diversity index of bacteria in the small intestine is much lower [34]. In addition, one study showed that microbiota in the ileum of pigs treated with antibiotics was significantly different from those in the cecum and colon [35]. It has also been found that bacteria in the ileum and the large intestine are spatially heterogeneous in antibiotic fed chicks [36]. There are many reasons for this phenomenon, including nutrient concentrations, pH and antibiotics, and physiological changes such as intestinal structure and host immunity [37]. We have observed significant differences in Observed_species, Shannon index, Chao1 index, and PD_whole_tree between the large intestine and duodenum (*p* < 0.05), which was consistent with the changes in intestinal microbiota diversity in weaned piglets after oral administration of antibiotics [38]. Thus, the FIS has very strong parameters to characterize the bacterial population of diarrhea and normal rabbits. Correspondingly, the bacterial diversity of the large intestine or small intestine is different among FIS, which our results similarly reflect. The diversity of the FIS was different between Dia and Con. In our study, we also found that the bacterial diversity of the FIS in Dia was lower than that in Con, which is consistent with a previous study in swine [39], indicating that the diarrhea of rabbits may be caused by the impairment of intestinal bacterial diversity after stopping antibiotics. It can be used as a research direction related to diarrhea in the future, and may become a target or biomarker of new microbial transplantation. 

Noteably, the results of PCoA based on weighted unifrac and AMOVA showed that there were significant differences in bacterial structures in the FIS of the two groups (*p* < 0.05), and the bacteria structure was also different between the small and large intestine, although not significantly between them (*p* < 0.05). At the same time, similar findings were found in similar segments. Therefore, the bacteria may have similar structural composition in similar intestinal segments or regions. The PCoA analyses revealed significant differences in the FIS between the Dia and Con, indicating that the intestinal microbiota of Dia changed. Consistent with previous studies, a PCoA of piglets showed that the intestinal microbiota microbiota of Con and Dia were also separated [40], indicating that the structure of intestinal microbiota between them had changed. 

When the balance between host and intestinal microbiota is broken, diarrhea will occur. In all intestinal content samples, a total of 21 bacterial phyla and 144 bacterial genera were detected. In line with other studies, our results revealed that the microbial communities of rabbits in the FIS contents of two groups were dominated by *Firmicutes*, *Proteobacteria*, and *Bacteroidetes* at the phylum level [41], and by *unidentified_ Clostridiales*, *Bacteroides*, and *lysinibacillus* at the genera of Dia; however, *Akkermansia* was the dominant bacteria in Con, which is partly consistent with previous results [41]. Furthermore, the relative abundance of each dominant phylum and genus varied greatly between the FIS of the two groups, suggesting that the intestinal microbial phylogeny was greatly affected in Dia. Any deviation from the normal composition of the intestine, termed as “microbial dysbiosis”, is characterized to include instability or a decrease in the relative abundance of *Firmicutes* [42]. This is consistent with our study, where the relative abundance of *Firmicutes* was lower in Dia, which also showed, once again, intestinal imbalance. The relatively high abundance of *Bacteroides* and *Proteobacteria* may be related to higher cellulose decomposition activity and proteolytic activity, respectively [43]. Our study found that the relative abundance of *Firmicutes* is lower in the large intestine than the small intestine of Dia. However, our findings were not consistent with the results of a previous study in which the relative abundance of *Firmicutes* in the porcine large intestine was higher than that in the small intestine, which the authors found may be the result of the intestine taking on some of the task of fat deposition [38].

Moreover, some studies also show that the relative abundance of *Firmicutes* in obese humans is high [44], which is also contrary to our results. Furthermore, previous studies of the intestinal microbiota of growing rabbits are also inconsistent with this, as they found a higher proportion of *Firmicutes* [45]. Therefore, compared with Con, the decrease in *Firmicutes* abundance in Dia may reduce fat content, thus affecting the quality and flavor of meat. To some extent, the characteristics of intestinal microbiota have gained increasing attention as a metabolic state and reflect the body composition of the host. We identified that the changes in these microbiomes may be due to the disturbance of normal metabolism and, therefore, the destruction of performance (e.g., body weight) in rabbits with diarrhea. Unfortunately, the need to measure fat content was not considered in our experiment, and more studies are needed. *Bacteroidetes* play a beneficial role in host health by interacting with the immune system and limiting the colonization of potential pathogens [46]. In addition, *Bacteroidetes* usually produce butyrate, which can maintain intestinal health. Previous studies have shown that antibiotic induced microbiome consumption can reduce the types of intestinal *Bacteroidetes*, mainly by reducing the reduction in SCFAs in the intestine, especially butyrate and stimulating bile acids, and changing normal metabolic homeostasis in the colon [47]. Notably, in our current study, the relative abundance of *Bacteroidetes* in the intestinal tract of the rabbits with diarrhea increased, suggesting that stopping the use of antibiotics helps to restore or increase the beneficial flora in the intestinal tract, which is worthy of further research. 

In Firmicutes, the dominant bacterial genera are concentrated in the small, but not the large, intestine and are only involved in the immune response of the large intestine, not the small intestine. *Clostridium butyricum*, a member of the *Firmicutes* genus *Clostridium*, mainly colonizes the cecum and colon, and also produces beneficial metabolites during metabolism, including various digestive enzymes, vitamin B, and SCFAs [48]. *Clostridium butyricum* can promote the αβ T cell receptors of intraepithelial lymphocytes and immunoglobulin. As causes of a cellular immune effect, these substances are key determinants of the extent of the body’s immune response to antigens or agent role. Studies have found that *Clostridium butyricum* can effectively reduce the inflammatory response and epithelial barrier damage in chickens [49]. However, inconsistently with our study, the *Clostridium butyricum* is more abundant in the small intestine of rabbits with diarrhea than in the large intestine. This may be due to the lack of this flora in the large intestine, which lacks beneficial metabolites and does not effectively reduce intestinal inflammation, leading to diarrhea. Moreover, our intestinal histology also found that there was inflammatory damage to the intestinal barrier. A certain amount of *Clostridium butyricum* can reduce oxidative damage and enhance the antioxidant capacity of mouse pathogenic models treated with toxin producing *Escherichia coli*, by activating the p62-Keap1-Nrf2 signal pathway and remodeling the intestinal microbial community [50]. We observed that the relative abundance of the dominant species members of *Firmicutes* in FIS was lower in Dia, consistent with previous finding on the gut microbiota of piglets with diarrhea [12]. Some members of *Firmicutes* can produce SCFAs through fermentation and the digestion of complex carbohydrates to regulate host immune system development and immune system tolerance [39]. However, in our study, *Clostridium_butyricum* was only detected in the Dia. In addition, it was not detected in the Con, and it’s relative abundance was relatively high in the duodenum of Dia. Therefore, our results are opposite to those of Con [51]; in our study, the abundance of *Clostridium* in the large intestine region is low, which may deplete key anti-inflammatory metabolites or other cell related immunomodulatory microflora [52], and the members of genus *Clostridium* may not participate in the immune response in the small intestine, resulting in intestinal diarrhea. 

In contrast to the *Firmicutes*, the relative abundance of the members of the phyla *Proteobacteria* increased in Dia, which was generally in line with a previous study in weaning piglets with diarrhea [39]. The most dominant bacterial genera of *Proteobacteria* included *Citrobacter*, *unidentified_Enterobacteriaceae*, *Vibrio*, and *Roseomonas*. With the exception of *Citrobacter*, all the other genera were concentrated in the large intestine rather than the small intestine. *Proteobacteria* is a major phylum of gram-negative bacillus, and the LPS endotoxin produced by *Proteobacteria* can enter the blood, reduce the number of intestinal barrier cells and intestinal permeability, and lead to chronic inflammation [53]. Previous studies have given that the imbalance in intestinal microbiota or the development of a host disease often arises from the continuous increase in *Proteobacteria* [54]. Compared with the Con, we found that the relative abundance of *Proteobacteria* in the Dia was increased, indicating that the proliferation of this harmful bacteria may disrupt gut homeostasis and cause inflammation by reducing gut barrier cell numbers and gut permeability. *Escherichia_coli* was not only the dominant bacterium in *Proteobacteria*, but its relative abundance was also higher in Dia than in Con. Previous studies have reported that *Escherichia_coli* can cause diarrhea and other infections in domestic animals [55]. Our finding was consistent with data from previous study [12], in which the fecal samples of weaned piglets that were naturally infected with *Escherichia_coli* were loose or watery, suggesting that *Escherichia_coli* infection was closely related to the diarrhea of the weaned piglets, and the intestinal microbiome diversity analysis showed that the relative abundance of *Escherichia_coli* was also high. In addition, it has been suggested that *Escherichia_coli* should be considered as a pathogenic or opportunistic pathogenic parasite in hosts. 

In addition, we also found *Terrisporobacter* as a biomarker in rabbits with diarrhea, which is related to a previous analysis of the human microbiome, with the relative abundance of this genus being increased in human autism patients [56]. Moreover, it is also related to inflammatory bowel disease, ulcerative colitis and indigestion antibiotic related diarrhea [57]. A previous study showed that the abundance of *Melainabacteria* was a significant difference between rats with small intestinal injury taking anti-inflammatory drugs and those without taking anti-inflammatory drugs, which was consistent with our study. In our present study, *Melainabacteria* became a biomarker in the jejunum of the Con, indicating that disruption of the microbiome could lead to diarrhea.

According to the prediction of the molecular function of FIS by Tax4Fun, at the second level of KEGG, most gut microbiome in the intestine mainly participate in amino acid metabolism, energy metabolism, glycan biosynthesis and metabolism, metabolism of cofactors and vitamins, signal transduction, and lipid metabolism. However, only the three metabolic pathways, including amino acid metabolism, energy metabolism and signal transduction, showed significant differences between Dia and Con (*p* < 0.05). The results have similarities to previous studies in fattening rabbits, in which KEGG pathways related to amino acid metabolism were abundant [58]. These functional changes should be related to the dynamics of the gut microbiota in the FIS. The amino acid metabolic pathway was significantly different among the FIS in the Dia, and the relative in the cecum was the highest, which was higher than that in the Con. The amino acid metabolic pathway is related to intestinal barrier function, oxidative stress in intestinal inflammation and the expression of anti-inflammatory and pro-inflammatory cytokines through a variety of signal pathways, and plays an important role in regulating intestinal homeostasis [59]. For example, indole propionic acid is an important amino acid metabolite produced by the microbiota in mammals, which can directly bind to specific receptors to strengthen the intestinal barrier. Previous studies have reported that *Clostridium* sporogenes from high abundance *Firmicutes* are related to indole propionic acid [60]. Interestingly, there is a significant difference, between the Dia and the Con, in the *Firmicutes* of the small intestine, and the Dia is more abundant, but the level of amino acid metabolism is just the opposite, indicating that the amino acid metabolic pathway is blocked, which may lead to the impairment of intestinal barrier function, intestinal inflammation, obstruction of the normal expression of anti-inflammatory and pro-inflammatory factors, and, finally, the imbalance of intestinal homeostasis. 

However, in our study, we found that the average relative abundance of *Firmicutes* in the Dia of the large intestine was lower than that in the Con, which may be due to the disordering of intestinal barrier function after the cessation of antibiotics due to the disordering of amino acid metabolism. At the same time, previous studies have also shown that the high abundance of bacteria involved in amino acid metabolism in *Firmicutes* can enhance intestinal mucosal immunity, and reduce intestinal oxidative stress and the diarrhea rate of piglets [61,62]. Therefore, the decrease in *Firmicutes* abundance in Dia may be due to the specific strains involved in amino acid metabolism reducing intestinal mucosal immunity and increasing intestinal oxidative stress levels. Intestinal microorganisms can obtain metabolic energy from the diet, such as metabolizing indigestible dietary fiber to convert into easily absorbed short chain fatty acids, which is one-tenth of the energy source and is responsible for about three-quarters of the energy metabolism of the intestinal epithelium. Therefore, the metabolic rate of SCFAs can determine the energy balance of the host [63]. It is worth noting that, compared with the Con in our study, we found that the carbohydrate metabolism function was also significantly different in the Dia, and the energy metabolism was also richer than the highest in the Con. Previous studies have shown that the large intestine cells of sterile mice lacking SCFAs are highly energy deficient, which is manifested in the reduced expression of key enzymes involved in fatty acid metabolism in mitochondria [64]. However, the addition of a SCFA to the colon of sterile mice can normalize this defect. Additionally, in our Dia, we found that the richness of *Firmicutes* and *Bacteroidetes*, which ferment nondigestible carbohydrates to produce a large number of SCFAs, was weaker than that of the Con. However, partially, among the FIS, the intestinal carbohydrate metabolism and energy metabolism in the small intestine are weaker in the Dia than in the Con, and the richness of *Firmicutes* and *Bacteroidetes* is also consistent with the functional level. It is possible that other low abundance microorganisms play an important role and have different functions in the large intestine. Furthermore, our results show that the metabolism of cofactors and vitamins, and Glycan biosynthesis and metabolism in the large intestine, is significantly different from that in the Con, and even metabolism plays a very important role, which is consistent with the research results of intestinal mucosa and lumen microbiota in pigs [65]. In addition, lipid metabolic activity was more abundant in the microbiota of small intestinal contents. This result suggests that microbial metabolic activity is more active in the large than the small intestine. The digestion of lipids in mammals is mainly concentrated in the small intestine, and the intestinal flora is involved in lipid metabolism [66]. Similarly, our results also indicate that lipid metabolism is more abundant in the small intestine. Amino acid metabolism in the small intestine is mainly related to specific transporters and enzymes in the host, while amino acid metabolism in the large intestine is more influenced by gut microbes, and the level of carbon source reaching the large intestine is lower; so, the microbes in the contents will utilize more nitrogen sources in order to break down undigested proteins and amino acids from the small intestine [67]. For herbivores, there are differences in the amount and type of carbohydrates in the large and small intestines, so there will be differences in the fermentative capacity of the microorganisms. Therefore, more rationally designed studies with a larger sample size are required to verify our finds. 

## 5. Conclusions

In summary, at phylum level, the dominant phyla *Firmicutes*, *Bacteroidetes* and *Proteobacteria* were significantly different in the FIS of the Dia and Con. In addition, the change in the relative abundance of the identified dominant microbiota, such as *Clostridium_butyricum,* and *Escherichia_coli*, may lead to the imbalance of microbiota and diarrhea due to the consumption of key anti-inflammatory metabolites, the destruction of carbohydrate homeostasis and the competition between superior and inferior microbiota. These results will be helpful for the pathogenesis and treatment of diarrhea in rabbits. 

## Figures and Tables

**Table 1:** The alpha diversity indexes of bacterial communities in the FIS of Hyplus rabbits.

Items	Group	Ce	Co	Du	Je	*p*-Value
Observed_species	Dia	75.50 ± 12.43 ^ab^	89.17 ± 17.94 ^a^	21.17 ± 5.712 ^c^	47.33 ± 14.54 ^bc^	0.0089
	Con	336.3 ± 98.01 ^a^	146.5 ± 11.88 ^b^	139.2 ± 8.750 ^b^	103.3 ± 10.04 ^b^	0.015
Shannon	Dia	3.620 ± 0.4404 ^a^	4.058 ± 0.5693 ^a^	1.534 ± 0.3940 ^b^	2.610 ± 0.5013 ^ab^	0.0065
	Con	6.594 ± 0.6705 ^a^	5.440 ± 0.1720 ^b^	5.828 ± 0.1382 ^ac^	5.033 ± 0.09586 ^b^	0.0351
Chao1	Dia	112.9 ± 21.11 ^a^	124.5 ± 27.23 ^a^	31.46 ± 9.863 ^b^	84.46 ± 27.47 ^ab^	0.0387
	Con	1304 ± 619.0 ^ab^	215.2 ± 23.58 ^b^	194.0 ± 21.56 ^a^	152.8 ± 17.90 ^ab^	0.0434
PD_whole_tree	Dia	22.59 ± 2.745 ^a^	22.31 ± 3.923 ^a^	7.078 ± 2.059 ^b^	13.46 ± 3.332 ^ab^	0.0048
	Con	66.85 ± 15.51 ^a^	34.62 ± 1.861 ^b^	32.52 ± 1.281 ^b^	25.47 ± 2.922 ^b^	0.0073

Data was obtained from four segments of intestinal contents of Hyplus rabbits (Con, *n* = 6; Dia, *n* = 6). The values are shown as the mean ± standard error (mean ± SE). Values in the same row with different superscripts (^a^, ^b^, ^c^) indicate significant differences among FIS (*p* < 0.05). Ce, cecum; Co, colon; Du, duodenum; Je, jejunum; observed_species; Chao1, Chao1 index; Shannon, Shannon index; PD_whole_tree.

## Data Availability

I confirm that the data is available.

## Supplementary Materials

The following supporting information can be downloaded at: https://www.mdpi.com/article/10.3390/microorganisms10050841/s1, Figure S1: Overview of Sequence data analysis. Rarefaction curves and rank abundance curves of lumen contents of four intestinal segments of Hyplus rabbits (Con, *n* = 6; Dia, *n* = 6). Rarefaction curves in (A) tend to be flat reflect that the sequencing data size is rational, and the x-axis of rarefaction curves indicates the sequences number selected randomly from samples and the y-axis indicates the observed species number (OTUs). In rank abundance curves (B), the x-axis indicates the order number ranked by the OTUs abundance while y-axis shows the relative abundance of OTUs. Observed species numbers (OUTs) were assigned at the 97% sequence similarity level. Co, colon; Ce, cecum; Du, duodenum; Je, jejunum; Figure S2: Distribution of bacterial phyla, genera, and species in the intestinal tract of Hyplus rabbits (Con, *n* = 6; Dia, *n* = 6). The circles in different colors represent different groups, corresponding to the legend on the left. The size of the sector represents the proportional size of the relative abundance of the group in the classification. The number below the taxonomic name represents the average relative abundance percentage of all groups in the classification. There are two numbers, the former represents the percentage of all species, and the latter represents the percentage of selected species. C, control group (Con, *n* = 6); E, diarrhea group (Dia, *n* = 6), Co, colon; Ce, cecum; Du, duodenum; Je, jejunum. Table S1: Number of sequence, Raw_reads, Clean_reads, Average Length (nt) and Effective; Table S2: The bacterial population structure can be determined by the analysis of AMOVA in the four intestinal segments of Hyplus rabbits (Con, *n* = 6; Dia, *n* = 6). C, control group (Con, *n* = 6); Dia, diarrhea group (Dia, *n* = 6); Co, colon; Ce, cecum; Du, duodenum; Je, jejunum, SE, standard error; Table S3: The bacterial population structure can be determined by the analysis of AMOVA in the four intestinal segments of Hyplus rabbits between Con and Dia; Table S4: Comparison of the 10 most abundant phyla in the four intestinal segment regions of Hyplus rabbits; Table S5: A total of genera were detected in four intestinal segment regions of Hyplus rabbits(Con, *n* = 6; Dia, *n* = 6); Table S6: Comparison of the genera across the four intestinal regions in the Hyplus rabbit(only genera with a relative abundance ≥5% in which at least one of the four segments were shown). Values in the same row with different superscripts (^a^, ^b^) indicate significant differences among FIS (*p* < 0.05).; Table S7: Predicted functions on the level 1 and 2 in the FIS (screened by the relative abundance of the genus in any one of the FIS at least ≥5%), except for significance, all the other data were presented as mean and standard error. Values in the same row with different superscripts (^a^, ^b^) indicate significant differences among FIS (*p* < 0.05).

## Abbreviations

FIS	Four intestinal segments
SCFAs	Short chain fatty acids
Dia	Diarrhea group
Con	Control group
16S rRNA	16S ribosomal RNA
IgA	Immunoglobulin A
