# Supplementary material for: Microbiomics Revealed the Disturbance of Intestinal Balance in Rabbits with Diarrhea Caused by Stopping the Use of an Antibiotic Diet
Source: Microorganisms. 2022 Apr 20;10(5):841. doi: 10.3390/microorganisms10050841 (PMC9145392; doi:10.3390/microorganisms10050841)
Supplement: Supplementary file 1 [file microorganisms-10-00841-s001.zip › Figures/figures.pdf]

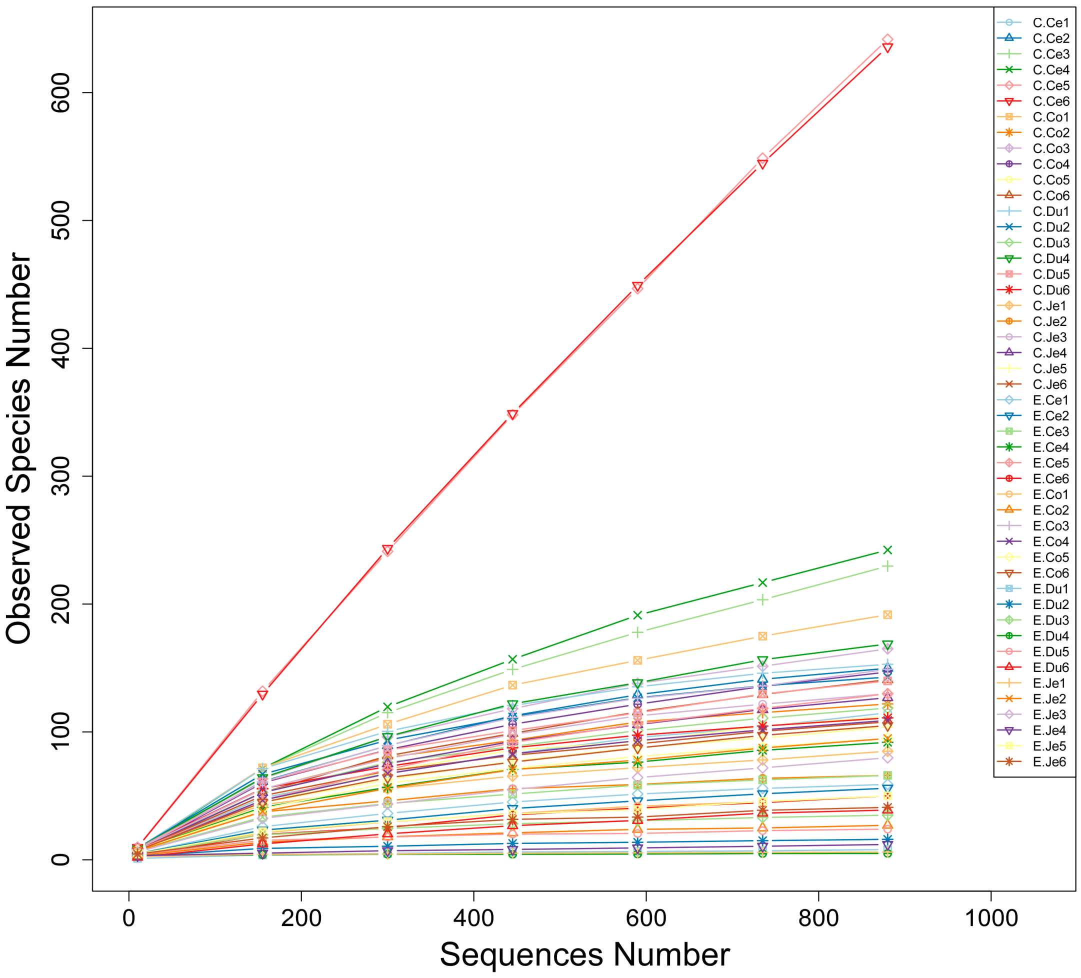

(A)

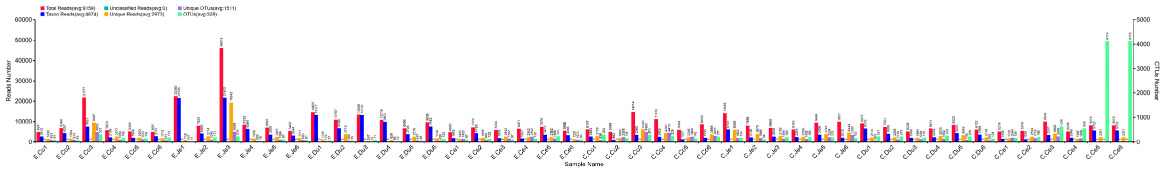

(B)

Figure S1. Overview of Sequence data analysis. Rarefaction curves and rank abundance curves of lumen contents of four intestinal segments of Hyplus rabbits (Con, n=6; Dia, n=6). Rarefaction curves in (A) tend to be flat reflect that the sequencing data size is rational, and the x-axis of rarefaction curves indicates the sequences number selected randomly from samples and the y-axis indicates the observed species number (OTUs). In rank abundance curves (B), the x-axis indicates the order number ranked by the OTUs abundance while y-axis shows the relative abundance of OTUs. Observed species numbers (OUTs) were assigned at the 97% sequence similarity level. Co, colon; Ce, cecum; Du, duodenum; Je, jejunum.

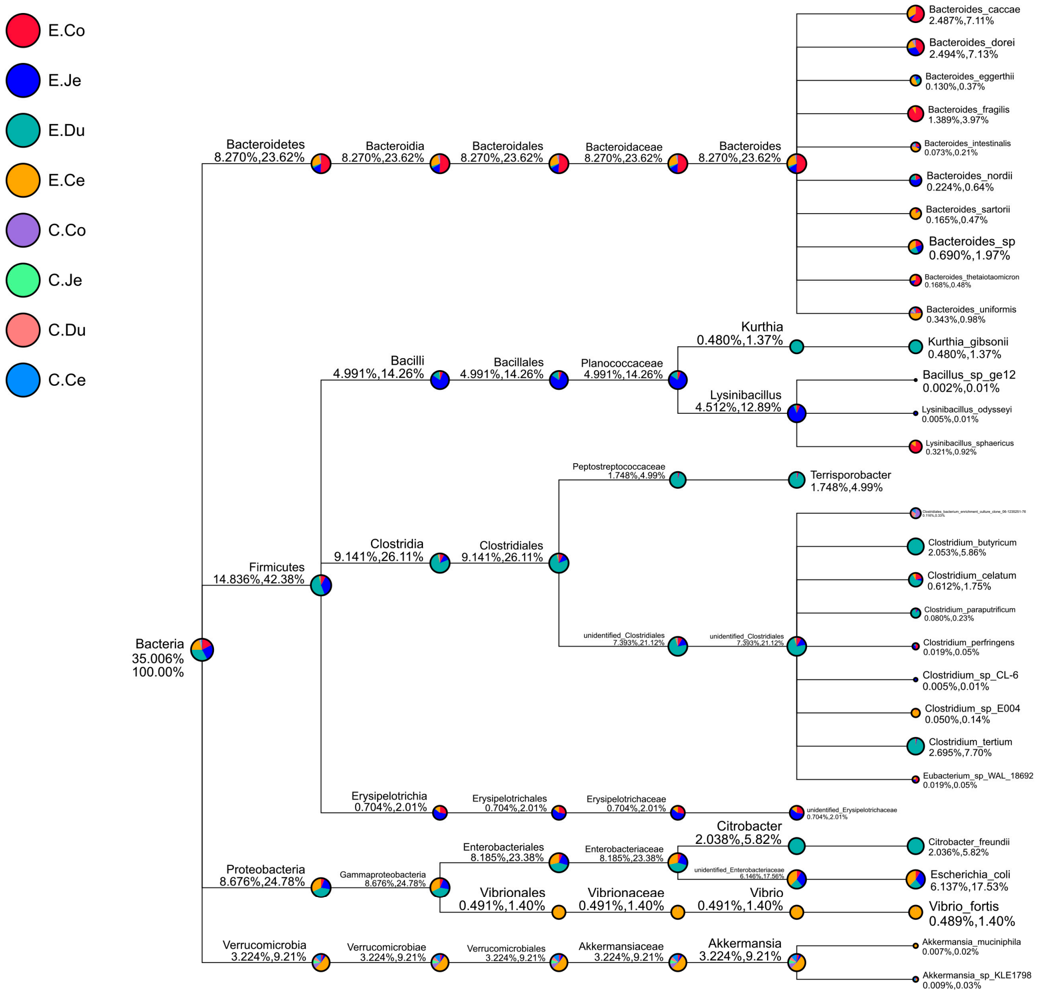

Figure S2. Distribution of bacterial phyla, genera, and species in the intestinal tract of Hyplus rabbits (Con, n=6; Dia, n=6). The circles in different colors represent different groups, corresponding to the legend on the left. The size of the sector represents the proportional size of the relative abundance of the group in the classification. The number below the taxonomic name represents the average relative abundance percentage of all groups in the classification. There are two numbers, the former represents the percentage of all species, and the latter represents the percentage of selected species. C, control group (Con, n=6); E, diarrhea group (Dia, n=6), Co, colon; Ce, cecum; Du, duodenum; Je, jejunum.
